# A Novel Approach for Development and Characterization of Effective Mosquito Repellent Cream Formulation Containing Citronella Oil

**DOI:** 10.1155/2014/786084

**Published:** 2014-10-14

**Authors:** Narayan Prasad Yadav, Vineet Kumar Rai, Nidhi Mishra, Priyam Sinha, Dnyaneshwar Umrao Bawankule, Anirban Pal, Arun Kumar Tripathi, Chandan Singh Chanotiya

**Affiliations:** ^1^Herbal Medicinal Products Department, CSIR-Central Institute of Medicinal and Aromatic Plants, P.O. CIMAP, Lucknow, Uttar Pradesh 226 015, India; ^2^Molecular Bioprospection Department, CSIR-Central Institute of Medicinal and Aromatic Plants, P.O. CIMAP, Lucknow, Uttar Pradesh 226 015, India; ^3^Plant Pathology Department, CSIR-Central Institute of Medicinal and Aromatic Plants, P.O. CIMAP, Lucknow, Uttar Pradesh 226 015, India; ^4^Laboratory of Aromatic Plants and Chiral Separation, CSIR-Central Institute of Medicinal and Aromatic Plants, P.O. CIMAP, Lucknow, Uttar Pradesh 226 015, India

## Abstract

Citronella essential oil (CEO) has been reported as an excellent mosquito repellent; however, mild irritancy and rapid volatility limit its topical application. It was aimed to develop a nonirritant, stable, and consistent cream of CEO with improved residence time on skin using an industrial approach. Phase inversion temperature technique was employed to prepare the cream. It was optimized and characterized based on sensorial evaluation, emulsification, and consistency in terms of softness, greasiness, stickiness, and pH. The optimum batch (B5) was evaluated for viscosity (90249.67 ± 139.95 cP), texture profile with respect to firmness (38.67 ± 0.88 g), spreadability (70.33 ± 0.88 mJ), and extrudability (639.67 ± 8.09 ± 0.1 mJ) using texture analyzer along with two most popular marketed products selected as reference standard. Subsequently, B5 was found to be stable for more than 90 days and showed enhanced duration of mosquito repellency as compared to CEO. HS-GC ensured the intactness of CEO in B5. Investigated primary irritation index (PII 0.45) positioned B5 into the category of irritation barely perceptible. The pronounced texture profile and stability of B5 with extended residence time and less PII revealed its potential application in industry and offered a promising alternative to the marketed products of synthetic origin.

## 1. Introduction

Mosquitoes such as* Aedes*,* Anopheles*, and* Culex* are a serious threat to the public health as they are known vectors for various protozoans, viruses, and bacteria which result in many life threatening diseases like malaria, filariasis, yellow fever, Japanese encephalitis, chikungunya, and dengue [1]. These vectors have been considered as a major obstacle to socioeconomic development of developing countries particularly in the tropical region [2]. Despite considerable efforts in recent years to control vector-borne diseases, malaria alone produces 250 million cases per year and 800,000 deaths including 85% children under five years (WHO, 2010) [3]. Therefore, the prevention of mosquitoes could be better than the cure of vector-borne disease. Hence, use of the mosquito repellents on exposed skin area is strongly recommended. Insect repellents usually work by providing a vapor barrier deterring the arthropod from coming into contact with the skin surface [1, 4]. Most of the commercial mosquito repellents are prepared using nonbiodegradable, synthetic chemicals like N, N-diethyl-3-methylbenzmide (DEET), dimethyl phthalate (DMP), and allethrin, which may lead to their higher exposure to the environment and, hence, the unacceptable health risks [5–7]. With an increasing concern on public safety, a renewed interest on the use of natural products of plant origin is desired because natural products are effective, environment friendly, biodegradable, inexpensive, and readily available [8, 9].

Currently, the US Environmental Protection Agency (US-EPA) has registered citronella, lemon, and eucalyptus oil as insect repellents due to their relatively low toxicity, high efficacy, and customer satisfaction. These are effective in the concentration range of 0.05% to 15% (w/v) alone or in combination with other natural or commercial insect repellents [10–12]. Citronella oil does repel mosquitoes and is required in its large amount to be effective due to the rapid volatility (evaporates too quickly from surfaces to which it is applied) and, hence, it would be unsafe for topical application because of its irritant nature (in the said concentration range) [13]. Formulating cream may ensure the avoidance of direct contact of the oil to skin and diminish the volatility, which would lead to the effective and safe (nonirritant) delivery of the oil for longer duration.

In-process quality control (IPQC) is a crucial phase in the manufacture of mosquito repellents. Some specific tests are performed at various time points in the manufacturing process to ensure that the finished products are consistent from run to run, remain effective over a long period, and are safe to use. Initially, raw materials are checked to ensure whether they meet the previously set specifications or not. Consequently, formulation of interest is tested on the basis of pH, specific gravity, and moisture content [13]. As far as development of cream (a semisolid formulation) is concerned, other unambiguous quality control parameter like texture profile need to be addressed appropriately in order to improve the stability, elegancy, and, hence user acceptance more deliberately.

In the present study, we are reporting a novel approach to develop a mosquito repellent cream formulation of citronella oil using phase inversion temperature technique and evaluating the cream by texture analyzer for firmness/hardness, spreadability, and extrudability. These parameters are considered as the quality measures of the cream characteristics and are desired for better consumer acceptance.

## 2. Materials and Methods

### 2.1. Materials

Citronella essential oil (CEO) was obtained from CSIR-Central Institute of Medicinal and Aromatic Plants, Lucknow, India. Stearic acid, cetyl alcohol, stearyl alcohol, potassium hydroxide, propylene glycol (PG), and glycerin were purchased from SD Fine Chem. Limited, Mumbai, India. Methyl paraben and propyl paraben were purchased from HiMedia Laboratories Pvt. Ltd., Mumbai, India. Double distilled Millipore water was used for formulation and evaluation. All the chemicals and reagents were of analytical grade and were used as received.

### 2.2. Methods

#### 2.2.1. Selection of Ingredients

Selection of ingredients was done according to their emulsification behavior. Various pilot batches of creams were prepared using a variety of oil phase ingredients. In order to get optimum consistency and property, several prototypes of cream formulation were prepared at different concentrations using finally selected ingredients, which are given in Table 1.

#### 2.2.2. Preparation of Cream

Table 2 summarizes the general heuristics of mixing sequence and technique used for the preparation of cream. While most of the heuristics are based on common practices, they can be derived from the basic knowledge of the underlying phenomenon of emulsion preparation. Detail design and prototyping are inevitably an iterative process. Depending on the product characterization and the performance test results, the product formula has to be revised. A number of standard performance tests are also available for a specific class of products. For example, in case of mosquito repellent cream, assessment of the mosquito repellency is required.

Each version of the prototype was inspected for its feel on application and stability (in terms of emulsification). If the prototype was found to be unstable, an improved version was prepared based on heuristics and experience. A prototype that passed the preliminary inspections (consistency and elegancy) underwent performance and stability tests. Further adjustments of the formula were made if the performance was not satisfactory. This iterative process continued until a satisfactory prototype was produced. For conciseness, the description below covers only 5 prototypes while many more were made in reality.

Phase inversion temperature method was applied for the preparation of cream. About 250 g cream sample was prepared in order to get a sufficient quantity for performing the various tests. The ingredients were categorized into two groups and mixed properly. The oil phase encompasses all the oil soluble ingredients, and the aqueous phase comprises the water soluble ingredients. The mixing procedure was developed based on the heuristics in Table 2. The oil phase was prepared by dispersing the oil soluble preservative, thickener, and stearic acid under mild heating and a stirring speed of 200 rpm using a hot magnetic plate stirrer (Magnetic Stirrer IKA RCT basic). All of the remaining components (except CEO) were added in the oil phase and heated to 65°C. The aqueous phase was prepared by mixing various aqueous soluble ingredients under gentle heating and stirring. Temperature of the aqueous phase was raised to 65°C. Just before mixing the two phases, the temperature of the oil phase was maintained at 55 ± 2°C followed by addition of CEO gently at a stirring speed of 200 rpm. The mixture was emulsified immediately by adding aqueous phase slowly from the wall of the container. For proper emulsification, the emulsion was maintained as such for 45 min at stirring rate of 800 rpm and 60 ± 2°C. The emulsified mixture was left for natural cooling. When the temperature of the emulsion reached 40°C, the organoleptic ingredients such as color and fragrance were added in to the emulsion with general mixing using a mechanical stirrer (Ika, Eurostar digital overhead stirrer). The cream was then kept in a bath sonicator for 5 min to remove any trapped bubbles and was then allowed to cool at room temperature. Since the pH value for cosmetic products is usually ranged from 5.5 to 8.0, the pH of the formulation was measured periodically and was maintained close to 6.7. In order to scale up the formulation, the same cream formula was used to prepare 2 kg of batch with IKA Eurostar WERKE Laboratory Reactor (Power Control visc P7). Cream was prepared successfully, packed in suitable container, and kept undisturbed at room temperature for further evaluation.

#### 2.2.3. Characterization and Optimization of Cream Formulation

Different prototypes of the cream formulation were characterized and optimized based on their aesthetic appearance, emulsification, pH, and consistency taking into account softness, greasiness, and stickiness. Sensorial observations including aesthetic appearance and consistency were assessed by twenty observers.

*Aesthetic Appearance*. The prepared formulation must be aesthetically elegant in terms of its physical appearance, color, odor, and texture. Therefore, the cream formulations were subjected to sensorial observations.

*Emulsification.* Improper emulsification generally brings about phase separation/cracking and precipitation showing instability. Therefore, the batches were observed for fine emulsification, which leads to an elegant preparation.

*pH Evaluation.* A definite amount of cream (100 mg) was weighed, diluted in distilled water, and mixed well. The pH of the cream was recorded using Digital pH Meter (Mettler Toledo). pH evaluation was carried out for all experimental formulations. The measurement was carried out in triplicate. 

*Consistency. *Each batch of the cream was evaluated for its consistency by examining its softness, greasiness, and stickiness. The formulation should be of uniform consistency which could spread and soften easily when stress is applied. It must also be nongreasy and nonsticky.

Depending upon the above findings, prototype B5 was selected as the optimized one (variation in prototypes is represented in Table 3). B5 was further subjected to assess* in-vivo* mosquito repellent study, primary irritation index, viscosity, stability studies, and texture analysis in terms of spreadability, firmness, and extrudability.

#### 2.2.4. Head Space-Gas Chromatography (HS-GC)

HS-GC analysis was carried out for CEO and mosquito repellent cream using Varian CP-3800 GC (Varian Associates, USA) with Combi Pal Head Space Gas Chromatography using DB-5 fused silica capillary column (30 m length × 0.25 mm internal diameter × 0.25 *μ*m film thickness) equipped with Flame Ionization Detector (FID). The oven temperature (60–240°C) was programmed at the rate of 3°C/min with a final hold of 2 min. Injector and detector temperature were 280°C. Hydrogen was used as a carrier gas at a rate of 1 mL/min and the split ratio was 1 : 40. Head space incubator was kept at 110°C for 15 min incubation time and 300 rpm. Head space syringe temperature was kept at 110°C with plunger speed of 250 *μ*L/sec and flush time of 8.5 sec. Head space volume was 900 *μ*L with plunger injector speed of 500 *μ*L/sec.

#### 2.2.5. Evaluation of Optimized Cream Formulation B5

*(1) Mosquito Repellent Activity*. Mosquito repellency was evaluated using a designer apparatus as reported by Tripathi et al. [14] with slight modification. The apparatus consists of a chamber A containing a smaller sized chamber B having copper wire mesh sheet on top and connected by a side tunnel to chamber C outside of chamber A. Chamber A was used to place the test sample, and chamber B was used to place the mosquitoes. Optimized formulation B5 (with 1%, 5%, and 10% CEO), placebo cream (B0), and crude CEO were investigated for mosquito repellent activity. For this study, the main criterion was migration of mosquitoes over fixed distance (from chamber B to C) after 2 h of sample application. A rabbit (anesthetized) was placed on the copper wire mesh surface of chamber B of the designer apparatus. Sixty adult female mosquitoes were released in chamber B. About 100 mg in case of each cream and 10 *μ*L of CEO were dispensed separately over a cardboard sheet of size (22 × 35 mm) and thickness (2.5 mm) equivalent to the commercially available mosquito mats. The treated cardboard sheet was placed in big chamber A of the designer apparatus for 2 h. After 0, 15, 30, 45, 60, 90, and 120 minutes of experimental duration, the number of mosquitoes present in both chambers (B and C) was counted, and the percentage of repellency was calculated from the following equation:

(1) $$\begin{array}{c} \%\text{Repellency}=\frac{\text{Total no. of mosquitos placed initially in chamber B}-\text{Number of mosquitoes present in chamber B}}{\text{Total No. of mosquitoes placed intially in chamber B}}\times 100. \end{array}$$

*(2) Texture Analysis*. B5 was evaluated for different texture parameters, namely, firmness, spreadability, and extrudability using different probes of CT3 Texture Analyzer (Brookfield Engineering Laboratories, USA) [15, 16]. Test parameters were selected as per individual test requirements recommended by Brookfield Engineering Laboratories, USA [17]. Data for the individual parameter was obtained from a graph generated by Texture Pro CT V1.3 Software. To assure the industrial application of B5, the same parameters were also analyzed for the two most popular marketed mosquito repellent products BM1 and BM2. Formulations were evaluated in triplicate and results are shown as mean ± S.E.M.

*(3) Viscosity Determination*. The viscosity of the B5 was measured along with placebo cream (B0) and two marketed formulations BM1 and BM2 using Brookfield Viscometer (DVLV-II+ pro model). The measurement was carried out at 25 ± 1°C, 10 rpm speed using spindle no. 61 in triplicate.

*(4) Skin Irritation Study*

*(a) Experimental Animals.* Adult, New Zealand white rabbits of either sex having body weight ~2.5 kg were received from Jeevanika, CSIR-Central Institute of Medicinal and Aromatic Plants, Lucknow, India. Animals were acclimatized to the experimental environment for 7 days before commencing the experiment (22 ± 5°C with humidity control and dark and light cycle of 12 h). They were provided* ad libitum* access to a commercial rabbit diet and drinking water. The experiment was carried out using OECD test guideline number 404 updated latest by 2002 [18] and the protocol (registration number 400/01/AB/CPCSEA, AH-2012-01) for this study was duly approved by Institutional Animal Ethics Committee (IAEC) following CPCSEA (Govt. of India) guidelines.

*(b) Experimental Protocol*. The back of each rabbit (*n* = 6) was clipped free of fur with curved scissor before 24 h of the application of the sample. The clipped area of skin was divided into two test sites of 1 inch square each. Normal saline was chosen as vehicle control and lactic acid (98% in distilled water) as standard irritant because it is a known irritant and its irritation grade is defined [19]. Rabbits were selected randomly and single test materials, namely, CEO 500 mg, cream base, B5 (corresponding to 500 mg of the CEO), and lactic acid 98%, were applied at a time on one test site of the animal against vehicle control. All the sites were covered by gauze and the back of the rabbit was wrapped with a nonocclusive bandage. After 4 h, the bandage was removed, sites were macropathologically examined for skin irritation, and the observation was repeated after 24, 48, and 72 h [20]. Skin reactions are graded separately for erythema and edema, each on a 0–4 grading scale. For erythema, 0 indicates no erythema, 1 very slight erythema/barely perceptible, 2 well-defined erythema, 3 moderate to severe erythema, and 4 severe erythema (beet redness) to slight eschar formation (injuries in depth). For edema, 0 indicates no edema, 1 very slight edema/barely perceptible, 2 slight edema (edges of area well defined by rising), 3 moderate edema (raised approximately 1 mm), and 4 severe edema (raised more than 1 mm and extending beyond the area of exposure). The primary irritation index (PII) was calculated as the arithmetical mean of the values of the six animals, that is, of the six patches with the same test-material. Test materials producing PII values as per OECD test guideline number 404 showed 0 as non-irritant, 0.04 to 0.99 as irritation barely perceptible, 1.00 to 1.99 as slightly irritant, 2.00 to 2.99 as mildly irritant, 3.00 to 5.99 as moderately irritant, and 6.00 to 8.00 as severely irritant [19]. Comparison between the mean values of PII of the experimental groups was made by one-way analysis of variance (ANOVA) followed by Tukey's post hoc test. All statistical analyses were performed using GraphPad Prism, Version 5.01 (GraphPad software. Inc., USA). The statistical significance of differences was accepted at the level of *P* < 0.05.

*(5) Stability Studies*. In accordance with International Conference on Harmonization (ICH) guidelines, stability analysis of optimized formulation (B5) was carried out. The optimized cream formulation (B5) was stored in well closed glass containers for a period of 90 days at 25°C temperature and 60% relative humidity in humidity chamber. At predetermined intervals, 0, 30, 60, and 90 days, samples were collected and their physicochemical evaluation parameters such as color, consistency, phase separation, texture analysis, and pH were evaluated.

*(6) Statistical Analysis*. Data are presented as mean ± standard error mean (SEM). Data for different groups were compared using one-way analysis of variance (ANOVA) followed by Tukey's post hoc test. All statistical analyses were performed using GraphPad Prism, Version 5.01 (GraphPad software. Inc., USA).

## 3. Results and Discussion

### 3.1. Characterization of Cream Formulation

Initial batches of formulated CEO cream were characterized for their elegancy, emulsification, and consistency based on sensorial evaluation. Suitable concentrations of the ingredients in the oil and aqueous phase were tested to develop cream to get desired emulsification, viscosity, consistency, spreadability, and stickiness. The cream was white in colour and opaque with homogeneous appearance.

The first prototype was observed to possess inappropriate elegancy, barely acceptable emulsification, very soft, and an acceptable degree of greasiness as well as stickiness. On increasing the concentration of cetostearyl alcohol from 2.0% to 4.0%, the second prototype (B2) was found to be very hard to spread. The pH was found to be changed from 5.5 to 6.1 by increasing KOH concentration from 0.7% to 0.8%. Therefore, third prototype (B3) was prepared using cetyl alcohol (2%) and stearyl alcohol (2%) instead of cetostearyl alcohol along with 7.5% glycerine, 2.5% propylene glycol, and 0.9% KOH. This prototype signified better emulsification and acceptable greasiness with pH 6.7 but consistency was found to be loose. Fourth prototype (B4) was prepared by increasing the concentration of stearic acid to 11.0% and decreasing the concentrations of cetyl alcohol and stearyl alcohol from 2.0% to 1.0%, which represented good emulsification, loose consistency, and nongreasiness of B4. In the fifth prototype (B5), increase in concentration of stearic acid to 13.0% and glycerine to 10.0% leads to the optimized batch with fairly good elegancy, emulsification, and consistency. The optimized batch possessed desired properties and was nongreasy and nonsticky favouring ease of its application to the skin surface. pH value of all the prepared batches was determined and found to be in the range of 5.5 to 6.7 (represented in Table 1). An increasing pattern of pH value was observed by increasing the concentration of KOH from 0.7 to 0.9%. Formulated batches with 0.9% KOH concentration were found to show pH~6.7 and were considered as the batches with favorable pH, that is, pH of skin (from 4.5 to 7.0).

### 3.2. HS-GC

**Figure 1:**
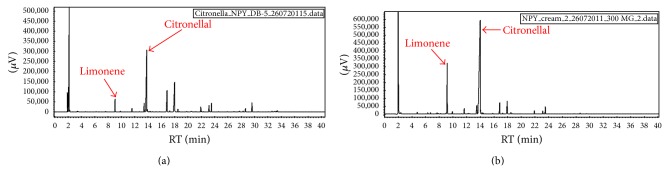
Head space-gas chromatographic (HS-GC) analysis of CEO and B5. GC chromatogram of (a) CEO (citronella essential oil) and (b) B5 (optimized batch of cream containing CEO). Peaks of chromatogram (b) represent the intactness of CEO in cream base.

The GC chromatogram of CEO is shown in Figure 1(a). The most abundant constituent of CEO in terms of relative percentage of total volatile oil was citronellal (40.659%) followed by geraniol (18.139%), citronellal (10.826%), limonene (3.941%), and linalool (1.154%), which is representing characteristic peaks of CEO. Chromatogram of CEO fabricated in cream base also reveals the same characteristic peaks (Figure 1(b)). Therefore, it represents the intactness of the oil in the cream base.

### 3.3. Evaluation of Cream Formulation (B5)

#### 3.3.1. Mosquito Repellency

**Figure 2:**
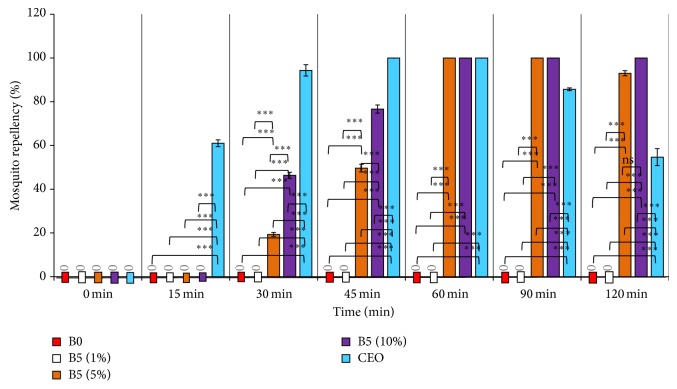
Mosquito repellent activity of CEO and B5. Graphical representation of mosquito repellent activity of different groups such as B0, cream base; CEO, citronella oil; B5 (1%), B5 (5%) and B5 (10%) are optimized prototypes with 1%, 5% and 10% of CEO. Data is presented as mean ± SEM (*n* = 3). Data was analyzed by one-way analysis of variance (ANOVA) followed by Tukey's post hoc test. ^***^ *P* < 0.001 is considered to be significantly different. ^ns^is reflecting the batches which are non-significantly different from one another.

Graphical presentation of percentage mosquito repellency by different groups is given in Figure 2. Cream base (B0) and B5 with 1% CEO were not able to repel the mosquitoes at any time point. At 15 min, only CEO showed significant change (^***^ *P* < 0.001) in the percentage of mosquito repellency (61%) than that of other groups. At 30 min of the study B5 (5%), B5 (10%), and CEO showed the significant mosquito repellency (^***^ *P* < 0.001); however, intergroup variation study showed that B5 (5%), B5 (10%), and CEO were also significantly different from each other. B5 (5%), B5 (10%), and CEO were found to have higher values of percentage repellency at 45 min than that found at 30 min, and CEO was showing 100% repellency. At 60 min, B5 (5%), B5 (10%), and CEO have reflected 100% repellency. At 90 min, B5 (5%) and B5 (10%) again showed 100% repellency; however, the value of the percentage repellency in case of CEO was observed to be declining significantly (∗∗∗*P* < 0.001). At 120 min, CEO showed further decline in the percentage repellency values. B5 (5%) also showed decline in the percentage repellency but was found to be nonsignificantly different from the value of B5 (10%), that is, 100%. This finding revealed that the B5 (10%) has enhanced the residence time of CEO over the surface. More than 50% mortality was also observed after 1 h in the case of pure CEO, while in the case of B5 (5%) and B5 (10%) it was seen after 2 h and 1.5 h, respectively.

#### 3.3.2. Texture Profile

**Figure 3:**
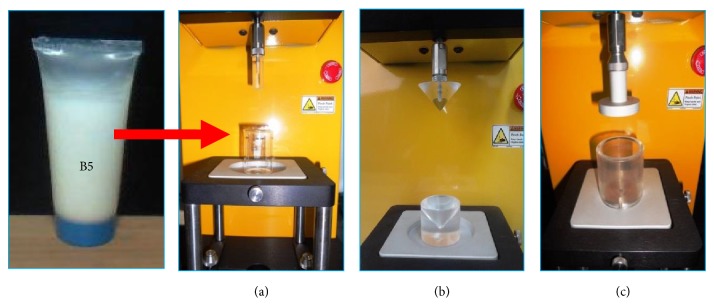
Pictorial presentation of texture analysis using CT3 Texture Analyzer. Photographs represent the assembly for the evaluation of* firmness* (photograph (a), with TA-10 probe and fixture TA-BT-KIT),* spreadability* (photograph (b), with male and female cone probes), and* extrudability* (photograph (c), with TA DEC (dual extrusion cell)) of the developed formulation (B5) and marketed formulation (BM1, BM2).

An Instrumental technology was employed to emulate human sensorial perception in terms of firmness, spreadability, and extrudability while evaluating semisolid preparations. These properties are collectively known as texture parameters of the formulation [18, 21, 22]. Pictorial representation of instrument assembly for texture analysis is given in Figure 3. Comparative texture profile of optimized batch (B5), along with two well-known marketed formulations (BM1 and BM2) and cream base (B0), is depicted in Table 4. Firmness is the maximum positive force required to deform the sample from finger. Firmness of B5 was found to be 38.67 ± 0.88 g, which is greater than that of BM2 (29.33 ± 0.33 g) and less than that of the cream base and BM1, which were recorded as 48.33 ± 0.67 g  and 54.33 ± 0.33 g , respectively. Hence, B5 is reasonably firm but softer than BM1. Value of the spreadability of B5 (70.33 ± 0.88 mJ)  was found to be less than that of B0 (80.67 ± 1.43 mJ), while BM1 and BM2 (95.67 ± 0.67 and 84.00 ± 1.00 mJ) showed higher spreadability among all. The lower value of spreadability indicates the lesser work done to spread the cream over the surface, which means formulation was easily spreadable by applying small amount of shear. Spreadability plays a considerable role in patient compliance and ensures uniform application of cream to a larger area of the skin [23]. The work done required to extrude B5 cream from tube was found to be 639.67 ± 9.28 mJ  while to extrude BM1, BM2, and B0 it was found to be 986.33 ± 8.99, 567.67 ± 9.74, and 741.67 ± 9.28 mJ, respectively. Technically certain amount of work would be done (mJ) to extrude the sample from a packaging tube uniformly [23, 24]. Higher extrudability of B5 than that of BM2 revealed that more work done was required to extrude B5 than that to extrude BM2. However, BM1 required slightly more work done to extrude from the tube. One of the selected marketed formulations was softer; however, another was a little bit harder. Since the developed cream formulation showed lesser spreadability and intermediate firmness and extrudability in comparison to two selected marketed formulations, it could be considered as the most aesthetic, consistent, and appealing. Texture analysis revealed that the CEO cream possessed fairly good spreadability, firmness, and extrudability, which are essential for its application and retention on the skin leading to a good consumer acceptability of the formulation.

#### 3.3.3. Viscosity

The viscosity of the optimized batch (B5) was found to be 90249.67 ± 139.95 cP  while viscosity of base, BM1, and BM2 was recorded to be 93160.67 ± 89.037, 100030.67 ± 36.70, and 82959.33 ± 35.83 cP, respectively (Table 4). Spreadability data supports the better acceptability of the developed cream at viscosity point of view, which is lying in between the range of the viscosity of the two marketed formulations, which will favor ease of application of B5 on skin surface.

#### 3.3.4. Skin Irritation Index

The results obtained from the primary skin irritation studies are listed in Table 5. There was significant difference (*P* < 0.001) in primary irritation index (PII) of vehicle control and standard irritant group, which indicates the irritation potential of lactic acid in the animals. The PII of the pure CEO was also significantly different (*P* < 0.001) from that of the vehicle control group on a scale of 0.0 to 8.0 affirming the fact that the above formulations are also causing significant irritation. The PII of cream comprising of CEO (B5) was found to be 0.45, which is non-significantly different from vehicle or placebo control group and positioned the prepared formulation into the category of irritation barely perceptible as per OECD guidelines (OECD, 2002) and make it appropriate for topical application. Furthermore, the PII of B5 was significantly less in comparison to the PII of CEO and standard irritant group. Skin irritation characteristic of CEO in terms of erythema and edema hampers its utility and acceptability for topical application. In our findings, CEO in cream (B5) demonstrated remarkable advantage over CEO in improving the skin tolerability, indicating its potential to improve patient acceptance and topical delivery as a mosquito repellent cream [20, 25].

#### 3.3.5. Stability Studies

Color, consistency, viscosity, texture profile, and pH of B5 were found to be consistent, and no separation was observed over the period of a 90-day study (Table 6), which revealed the reproducibility of the physical and chemical parameters which ensures the consistent quality of the developed cream formulation.

## 4. Conclusion

Safe and effective mosquito repellent cream formulation of CEO was successfully developed. Fabrication of CEO in cream base reduced the irritancy and volatility and exhibited potent repellent action. It reduced primary irritation index that is from mild irritant (PII2.035) to irritation barely perceptible (PII0.45) that offer safe delivery of the oil over the skin. Exploiting CT3 Texture Analyzer for the cream characterization in terms of firmness, spreadability, and extrudability has been recognized as a promising tool to the formulation development. This instrumental technique helped to develop an aesthetic and consistent cream. Favourable texture profile along with viscosity has ensured the quality and stability of the cream (B5) at par the marketed formulation.

## Figures and Tables

**Table 1:** Prototypes of mosquito repellent cream.

Ingredient	HLB value	Properties	Prototype (wt%)				
			1	2	3	4	5
Oil phase							
Stearic acid	15.0	Emulsifier and oil base	9	9	9	11	13
Cetostearyl alcohol	15.5	Emulsifier/stiffener	2	4	—	—	—
Cetyl alcohol	15	Stiffening agent, thickener	—	—	2	1	1
Stearyl alcohol	10–12	Coemulsifier, thickener	—	—	2	1	1
Citronella oil	12.6	Mosquito repellent	10	10	10	10	10
Aqueous phase							
Glycerin	—	Humectant, plasticizer	5	5	7.5	7.5	10
Propylene glycol (PG)		Humectant, plasticizer	2.5	5	2.5	—	—
Potassium hydroxide (KOH)	—	Saponifier	0.7	0.8	0.9	0.9	0.9
Distilled water	—	—	q. s.	q. s.	q. s.	q. s.	q. s.
Preservatives							
Propyl paraben	—	Oil soluble preservative	0.05	0.05	0.05	0.05	0.05
Methyl paraben	—	Water soluble preservative	0.1	0.1	0.1	0.1	0.1
Organoleptic ingredient							
Colours	—	Coloring pigment	—	—	—	—	q. s.
Fragrances	—	Fragrances and perfumes	—	—	—	—	q. s.

**Table 2:** Heuristics for formulation development.

Mixing sequence (i) Prepare aqueous phase and oil phase into separate containers before mixing. (ii) Add oil soluble preservative to oil phase and water soluble preservative to water phase. (iii) To make O/W emulsion, keep the amount of aqueous phase higher. (iv) To make W/O emulsion, keep the amount of oil phase higher than or equal to that of aqueous phase. (v) Add aqueous phase to oil phase through wall of the container to ensure little loss of two phases. (vi) Stir emulsion at constant temperature (from 55 to 65°C) for 45–60 min for proper emulsification. (vii) After proper emulsification, add heat-sensitive ingredients such as color, pH adjuster, fragrances, and perfumes at lower temperature (below 40°C).	

Preparation of aqueous phase (i) Aqueous phase ingredients such as glycerin and water soluble preservative should be predissolved with a sufficient quantity of distilled water. (ii) The emulsifiers should be added as the last part of aqueous phase.	

Preparation of oil phase (i) Add liquid emollients to solids emollients and melt them using gentle heating. (ii) Add essential oil (CEO) at 55°C into oil phase just before mixing the two phases.	

Author recommendations (i) Homogenizer/mixer with adjustable stirring speed is recommended to use for the bench scale experiment and also for the production scale. (ii) Keep the stirring speed of mixer or high-shear homogenizer below 2,000 rpm to avoid breaking down the carbon chains, which leads to foaming of emulsion and causes instability. (iii) To get proper emulsification during the addition of two phases, stirring speed should be kept at 200–400 rpm to avoid bubble formation and after that speed should be maintained near 800–1000 rpm. (iv) After addition of two phases, emulsion should be stirred for 45–60 min. (v) Ultrasonication (using bath sonicator) is recommended in the case of air entrapment.	

**Table 3:** Optimization of mosquito repellent cream.

Parameters	Prototype 1		Prototype 2		Prototype 3		Prototype 4		Prototype 5	
	Results	Changes	Results	Changes	Results	Changes	Results	Changes	Results	Changes
Elegancy	Poor	—	Acceptable	—	Acceptable	—	Good	—	Good	—
Emulsification	Acceptable	—	Acceptable	Change emulsifier	Good	—	Good	—	Good	—
pH	5.5	(+) KOH	6.1	(+) KOH	6.7	—	6.7	—	6.7	—
Consistency										
Softness^@^	Very soft/not-acceptable	(+) Cetostearyl alcohol	Hard/not-acceptable	(−) Cetostearyl alcohol (+) Stearyl and cetyl alcohol	Very soft/not-acceptable	(+) Stearic acid	Soft/acceptable	(+) Stearic acid	Soft/acceptable	—
Greasiness^@^	Acceptable	—	Acceptable	(−) PG	Acceptable	(−) PG	Acceptable	—	Non-greasy/Acceptable	—
Stickiness^@^	Acceptable	—	Sticky/not-acceptable	(−) Cetostearyl alcohol	Sticky/not-acceptable	—	Sticky/not-acceptable	(−) PG	Non-sticky/Acceptable	—

^@^Acceptable/not-acceptable, PG: propylene glycol, and KOH: potassium hydroxide.

**Table 4:** Texture profile and viscosity of marketed formulations and B5 cream.

Parameters	B0	B5	BM1	BM2
Texture profile				
Spreadability (mJ)	80.67 ± 1.43	70.33 ± 0.88	95.67 ± 0.67	84.00 ± 1.00^ns^
Firmness (g)	48.33 ± 0.67	38.67 ± 0.88	54.33 ± 0.33	29.33 ± 0.33
Extrudability (mJ)	741.67 ± 9.28	639.67 ± 8.09	986.33 ± 8.99	567.67 ± 9.74
Viscosity (cP)	93160.67 ± 89.04	90249.67 ± 139.95	100030.67 ± 36.70	82959.33 ± 35.83

B0, cream base; B5, optimized formulation; BM1 and BM2 are marketed formulations first and second, respectively. Data is presented as mean ± SEM (*n* = 3). Data was analysed by one-way analysis of variance (ANOVA) followed by Tukey's post hoc test. All the groups are significantly different (^***^ *P* < 0.001) from each other. ^ns^is reflecting the non-significant difference among spreadability of B0 and BM2.

**Table 5:** Erythema and edema scores for skin irritation index.

Sample	Reactions	4 h						24 h						48 h						72 h						PII	Results
		1	2	3	4	5	6	1	2	3	4	5	6	1	2	3	4	5	6	1	2	3	4	5	6		
Saline																											
—	Erythema	0	0	0	0	0	0	0	0	0	0	0	0	0	0	0	0	0	0	0	0	0	0	0	0	0.0	Nonirritant
	Edema	0	0	0	0	0	0	0	0	0	0	0	0	0	0	0	0	0	0	0	0	0	0	0	0		
Base																											
Test site	Erythema	0	0	0	0	0	0	0	0	0	0	0	0	0	0	0	0	0	0	0	1	0	0	1	0	0.0416^ns^	Irritation barely perceptible
	Edema	0	0	0	0	0	0	0	0	0	0	0	0	0	0	0	0	0	0	0	0	0	0	0	0		
Control site	Erythema	0	0	0	0	0	0	0	0	0	0	0	0	0	0	0	0	0	0	0	0	0	0	0	1		
	Edema	0	0	0	0	0	0	0	0	0	0	0	0	0	0	0	0	0	0	0	0	0	0	0	0		
Standard irritant																											
Test site	Erythema	2	2	3	2	2	2	2	2	2	2	2	3	2	2	2	1	2	1	1	1	1	2	1	1	3.833∗∗∗	Moderately irritant
	Edema	4	4	4	4	4	3	4	4	3	2	2	3	3	2	1	2	1	1	1	1	2	2	1	1		
Control site	Erythema	0	1	0	1	0	1	0	0	0	0	0	0	1	0	0	0	0	0	0	0	0	0	0	0		
	Edema	0	1	0	1	0	1	0	0	0	1	0	0	1	0	0	0	0	0	0	0	0	0	0	0		
CEO																											
Test site	Erythema	1	1	1	1	1	1	1	1	1	2	2	2	2	2	3	3	3	3	3	3	3	3	3	4	2.035^∗∗∗a^	Mildly irritant
	Edema	0	0	0	0	0	0	0	0	1	0	0	0	0	0	0	0	0	0	0	0	0	0	0	0		
Control site	Erythema	1	0	0	0	0	0	0	0	1	0	0	0	0	1	0	0	0	0	0	0	0	0	0	0		
	Edema	0	0	0	0	0	0	0	0	0	0	0	0	0	0	0	0	0	0	0	0	0	0	0	0		
B5																											
Test site	Erythema	0	0	2	1	1	1	1	1	1	1	0	2	0	1	0	1	0	1	0	0	0	0	0	0	0.45^ns/a^	Irritation barely perceptible
	Edema	0	0	2	0	0	0	1	0	1	1	0	1	0	0	0	0	0	0	0	0	0	0	0	0		
Control site	Erythema	0	1	0	1	0	1	0	0	1	0	1	0	1	0	1	0	0	0	0	0	0	0	0	0		
	Edema	0	1	0	0	0	0	0	0	1	0	0	0	0	0	0	0	0	0	0	0	0	0	0	0		

For erythema: 0 = no erythema, 1 = very slight erythema (barely perceptible), 2 = well-defined erythema, 3 = moderate to severe erythema, and 4 = severe erythema (beet redness) to slight eschar formations.

For edema: 0 = no edema, 1 = very slight edema (barely perceptible), 2 = slight edema (edges of area well defined by definite rising), 3 = moderate edema (raised approximately 1 millimeter), and 4 = severe edema.

Evaluation of primary irritation index (PII): 0.00: no irritation, 0.04–0.99: irritation barely perceptible, 1.00–1.99: slight irritation, 2.00–2.99: mild irritation, 3.00–5.99: moderate irritation, and 6.00–8.00: severe irritation.

Data was analyzed by one-way analysis of variance (ANOVA) followed by Tukey's post hoc test. ^ns^non-significantly different from saline control, ^***^ *P* < 0.001, when standard irritant and test control (cream base, CEO and B5) were compared with vehicle control. ^a^ *P* < 0.001, when B5 and CEO were compared with standard irritant.

**Table 6:** Stability study of B5 cream.

Formulations characteristics (days)	B5			
	0	30	60	90
Color^@^	Acceptable	Acceptable	Acceptable	Acceptable
Separation^@^	No	No	No	No
Consistency^@^	Acceptable	Acceptable	Acceptable	Acceptable
Texture profile∗				
Spreadability (mJ)	70.33 ± 0.88	70.33 ± 1.20	71 ± 1.53	70 ± 1.73
Firmness (g)	38.67 ± 0.88	40.00 ± 1.15	40.67 ± 0.88	41.3 ± 0.33
Extrudability (mJ)	639.67 ± 8.09	635.00 ± 13.89	646.67 ± 4.18	635.00 ± 8.14
Viscosity (cP)∗	30150.7 ± 81.5	90249.67 ± 139.95	90149.67 ± 229.90	90583.00 ± 58.39
pH∗	6.63 ± 0.02	6.79 ± 0.02	6.73 ± 0.01	6.77 ± 0.02

^@^Based on sensorial evaluation.

∗Tolerance of stability after one freeze/thaw cycle. Stable: change < 10%, acceptable: 10% < change < 20%, unstable: 20% < change < 40%, and unacceptable: change > 40%.

Data is presented as mean ± SEM (*n* = 3).
